# 

**Published:** 1881-12

**Authors:** 


### CONTENTS:

PAGE.

ORIGINAL COMMUNICATIONS:

I. Address delivered before the Chicago Gynæcological Society. By De Laskie Miller, M.D., of Chicago .......... 562

II. A Contribution to the Minute Anatomy of the Skin. By C. Heitzmann, M.D., of New York. (Illustrated) .......... 575

III. Notes on Crustacea in Chicago Water Supply, with some Remarks on the Formation of the Carapace. By E. A. Birge, Univ. of Wisconsin, Madison, Wis. (Illustrated) .......... 584

IV. Studies in the Medical Botany of Southern Illinois. II. By J. M. G. Carter, M.D. 590

V. Malarial Keratitis. By F. C. Hotz, M.D., of Chicago .......... 578

CLINICAL REPORTS:

VI. Fistula upon the Face. By Eugene S. Talbot, M.D., D.D.S. .......... 604

PAGE.

SOCIETY REPORTS:

VII. Chicago Medical Society .......... 607

VIII. Michigan State Board of Health .......... 609

IX. American Dermatological Association. Official Report of the Proceedings, by the Secretary, Dr. Arthur Van Harlingen, of Philadelphia. (Concluded) .......... 612

FOREIGN CORRESPONDENCE:

X. Letter from London .......... 635

DOMESTIC CORRESPONDENCE:

XI. New Medical Colleges .......... 639

XII. Letter from Prescott, Wis .......... 641

XIII. Medical Electricity .......... 643

REVIEWS AND BOOK NOTICES .......... 650

ORIGINAL TRANSLATIONS .......... 652

SELECTIONS .......... 658

ITEMS .......... 603, 605, 634, 649, 657, 670, 671

ANNOUNCEMENTS FOR THE MONTH .......... 672

NOTICE TO CORRESPONDENTS .......... advts.

